# Development of a sheep model of atrioventricular block for the application of novel therapies

**DOI:** 10.1371/journal.pone.0229092

**Published:** 2020-02-10

**Authors:** Melad Farraha, Juntang Lu, Ivana Trivic, Michael A. Barry, James Chong, Saurabh Kumar, Eddy Kizana

**Affiliations:** 1 Sydney Medical School, The University of Sydney, Australia; 2 Center for Heart Research, The Westmead Institute for Medical Research, Sydney, Australia; 3 Department of Cardiology, Westmead Hospital, Sydney, Australia; Mount Sinai School of Medicine, UNITED STATES

## Abstract

**Introduction:**

Sheep have been adopted as a pre-clinical large animal for scientific research as they are good models of cardiac anatomy and physiology, and allow for investigation of pathophysiological processes which occur in the large mammalian heart. There is, however, no defined model of atrioventricular block in sheep to allow for pre-clinical assessment of new cardiac treatment options. We therefore aimed to develop an adult sheep model of atrioventricular block with the focus on future novel applications.

**Methods and results:**

We utilized six sheep to undergo two procedures each. The first procedure involved implantation of a single chamber pacemaker into the right ventricular apex, for baseline assessment over four weeks. The second procedure involved creating atrioventricular block by radiofrequency ablation of the His bundle, before holding for a further four weeks. Interrogation of pacemakers and electrocardiograms determined the persistence of atrioventricular block during the follow up period. Pacemakers were inserted, and atrioventricular block created in 6 animals using a conventional approach. One animal died following ablation of the His bundle, due to procedural complications. Four unablated sheep were assessed for baseline data over four weeks and showed 5.53 ± 1.28% pacing reliance. Five sheep were assessed over four weeks following His bundle ablation and showed continuous (98.89 ± 0.81%) ventricular pacing attributable to persistent atrioventricular block, with no major complications.

**Conclusion:**

We have successfully developed, characterized and validated a large animal model of atrioventricular block that is stable and technically feasible in adult sheep. This model will allow for the advancement of novel therapies, including the development of cell and gene-based therapies.

## Introduction

Over the past two decades, the development of biological pacemakers via cell and gene-based therapies has progressed from proof-of-concept studies in small animal models [1–3] to experimental therapeutic strategies for the treatment of human diseases. The advancement to clinical trials, however, requires extensive pre-clinical assessment in large animal models to validate the safety and efficacy of the chosen approach. To date, only a few studies for the development of biological pacemakers have progressed to large animals, namely dog [4–6] and pig [7–9] models, each with their strengths and weaknesses.

Whilst studies in dog and pig models have laid the foundation for the advancement of biological pacemakers, we believe that sheep (*Ovis aries*) represent a more clinically relevant model for cardiovascular research. They represent good models of cardiac anatomy and physiology, while allowing for greater scrutiny of the pathophysiological processes that occur [10]. Further, sheep do not grow as fast and as large as pig models, allowing for easier handling of the animals and better standardisation of the data.

There is, however, no well-defined adult, sheep model of atrioventricular block (AVB) created using clinically relevant tools and techniques, for use in the assessment of cell and gene therapy-based treatments. Large animal models are superior to rodent models of bradyarrhythmia, even though a recent study reported a rat model of chronic and complete AV block displayed key clinical indices of severe bradycardia [11]. A study successfully developed a paediatric sheep model of complete heart block using an atypical approach from the left side of the heart [12], anticipating its use in the advancement of cell-based and other innovative treatments to repair complete heart block in children–this approach however is not practical in the adult heart. Two other studies utilized radiofrequency (RF) catheter ablations as ways of producing AVB for the assessment of bradycardia-induced spontaneous tachyarrhythmias and sudden death in conscious sheep following chronic myocardial infarction [13] and to assess the feasibility of using very high frequency current with a newly designed generator, to examine the effects of RF ablations on the AV junction and ventricular myocardium in sheep [14]. Although excellent studies, they did not focus on creating a clinically relevant large animal model of AVB while confirming its persistence in the follow up period.

To maintain clinical relevance of our experimental design, we performed procedures typically employed in the treatment of human diseases, using equipment designed for human use. These procedures included fluoroscopically guided pacemaker generator and active-fixation lead implantation, fluoroscopically and intracardiac echocardiography (ICE) guided endocardial RF ablation of the His bundle as well as electrocardiogram (ECG) monitoring and pacemaker device interrogation.

Creation and characterisation of a model of permanent AVB is a requirement in the development and assessment of biological pacemakers as incomplete AVB could confound findings following a novel treatment approach. We therefore aimed to develop, characterize and validate an adult sheep model of AVB to meet this stringent requirement for the assessment of novel cell and gene therapy-based approaches to biological pacemakers.

## Materials and methods

### Experimental design

**Fig 1:**
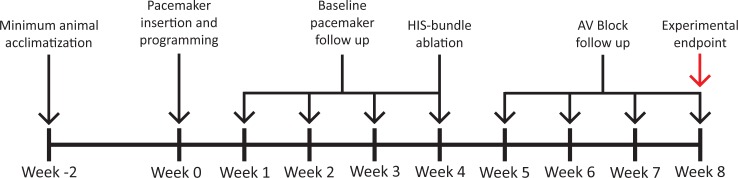
Experimental timeline depicting the stages at which the sheep were subjected to pacemaker insertion, His bundle ablation and monitoring for data collection.

Animals were acclimatized for a minimum of 2 weeks before commencing experimental work according to the timeline outlined in (Fig 1). Animals randomly received either an Insync 8040 (Medtronic, MN, USA), Eluna 8 SR-T (Biotronik, Berlin, Germany) or Iperia 7 DR-T (Biotronik, Berlin, Germany) implantable device and were monitored over 4 weeks for collection of baseline data. At the 4-week timepoint, animals underwent His bundle ablation, after which they were monitored for a further 4 weeks.

### Animal handling

All animal studies were approved by the Western Sydney Local Health District Animal Ethics Committee (Protocol Number: 4271.06.17) and conducted in accordance with the NSW Department of Primary Industries, Animal Welfare Branch Guidelines for the Care and Use of Laboratory Animals under the Animal Research Act 1985.

Adult, male, castrated, merino cross sheep, (*n* = 6; age, ~1 year; weight, 63.1 ± 5.6 kg), were used in this study and were fasted for 24 hours before surgery to prevent emetic related complications. Sedation was induced with an intramuscular (IM) injection of xylazine (0.04 mg/kg, [Ilium, Troy Laboratories, NSW, Australia]). Following sedation, anaesthesia was induced with intravenous (IV) injection of propofol (5 mg/kg, [Braun, Kronberg Im Taunus, Germany]) followed by endotracheal intubation. Anaesthesia was maintained with 2–5% isoflurane (Aerrane, Baxter, IL, USA) mixed with 100% oxygen via mechanical ventilation. Wool was shorn at sites requiring access on the sheep. Surgical sites were prepared using standard sterile procedures and intravascular access was obtained percutaneously at the right femoral artery (invasive blood pressure monitoring), right femoral vein (catheter access), left internal jugular vein (anaesthesia induction and intravenous fluids) and right internal jugular vein (pacemaker lead implantation). Following vascular access, a bolus dose of Heparin (80 IU/kg, [Pfizer, NY, USA]) was administered to decrease the risk of clot formation. Heart rate, arterial oxygen (O_2_) saturation, end-tidal carbon dioxide output (ETCO_2_), surface ECG, invasive arterial blood pressure, respiration rates and volume and consciousness were monitored throughout all procedures. Following all procedures, catheters and vascular access were removed, anaesthesia ceased, and animals extubated and stabilized before being recovered to the appropriate holding pens. Benacillin (2 mg/kg, [Ilium, Troy Laboratories, NSW, Australia]) and buprenorphine (0.0025 mg/kg, [Reckitt Benchiser, England, UK]) were administered IM at the conclusion of each procedure for antibiotic prophylaxis and analgesia, respectively.

### Pacemaker implantation

Following anaesthesia, each animal was placed in a supine position with its limbs gently secured to the procedure table. The front left limb was secured in a cranial flexion position and the front right limb was secured in a caudal extension position to allow access to the neck for pacemaker insertion. The right internal jugular vein was identified, and a subcutaneous pocket created at the base of the neck. The pocket was created at the beginning to minimize disturbance to the lead and generator once implanted. Once the pocket was created, the right internal jugular vein was cannulated with a 9 French (Fr) sheath. Depending on the pacemaker device (Table 1), a corresponding ventricular lead was positioned at the right ventricular (RV) apex using fluoroscopic (Syngo artis zee VC14J, Siemens, Berlin, Germany) and ICE (Acuson sequoia C512, Siemens, Berlin, Germany) guidance using a percutaneous lead introducer (Safe Sheath II, Oscor, NY, USA) and appropriate stylets (Medtronic, MN, USA). The lead position was further refined by testing threshold pacing capture and sensed R-wave values using the corresponding pacemaker programmer–Model 2090 (Medtronic, MN, USA) or PSW 1602.A/1 (Biotronik, Berlin, Germany). Leads were then secured in place and connected to the pacemaker device which was set to pace in VVI mode at a rate of 60 beats per minute (bpm). The device was then secured in the subcutaneous pocket and the incision sutured closed using standard surgical techniques.

**Table 1:** Manufacturer and model details relating to pacemaker devices and leads.

Animal Number	Device Manufacturer	Device Model	Lead Manufacturer	Lead Model	Description	Fixation and length
M8, M9, M11	Medtronic	Insync 8040	Biotronik	Solia S53	Steroid eluting lead	Active– 53cm
M12	Biotronik	Eluna 8 SR-T	Biotronik	Solia S53	Steroid eluting lead	Active– 53cm
M10, M13	Biotronik	Iperia 7 DR-T	Biotronik	Protego ProMRI S 65/18	Quadripolar ICD lead	Active– 65cm

### His bundle ablation

The right femoral vein was cannulated with 9 and 12 Fr sheaths. An ICE catheter was advanced, via the 12 Fr sheath, to the right atrium to allow for visualisation of the tricuspid valve annulus and the location of the His bundle at the mid-atrial septum, anterior to the coronary sinus. Under fluoroscopic guidance, a 7 Fr non-irrigated, Safire, Bi-directional ablation catheter (St. Jude Medical, USA) was advanced, via the 9Fr sheath to the region of the His bundle. The ablation catheter was connected to both a Stockert ep shuttle RF generator (ST-0597, Biosense Webster, USA) and Cardiolab EP multichannel ECG recording system (GE Healthcare, USA). The His bundle was located using bipolar electrograms from the ablation catheter proximal pair showing the typical His electrogram. Once located, RF generated energy was delivered to the tissue for 10–60 seconds at 50–60 watts with a temperature limit of 60°C. A lifePak 12, biphasic defibrillator (Medtronic, MN, USA) was used to defibrillate/cardiovert ventricular fibrillation (VF) or ventricular tachycardia (VT) as required. A successful ablation was confirmed by complete AVB for a minimum of 20 minutes, as well as pacemaker dependence as evident on the ECG and pacemaker interrogation. Following creation of AVB, the pacemaker rate was increased to 80 bpm to align with the intrinsic heart rate of sheep.

### Pacemaker interrogation for collection of follow up data

Devices were interrogated the day after each procedure to confirm pacemaker functionality, and weekly to collect data on persistence of AVB and pacemaker dependence. To perform the interrogations, animals were brought into internal holding pens from the outside paddock and the programming device’s magnetic interrogator placed over the pacemaker device at the base of the animal’s neck. Data collection included: pacing percentage, device status, pacing parameters, waveform segments and sensing data. Once complete, the animals were promptly returned to the paddock.

### Statistical analysis

All data is presented as mean ± standard error of the mean. Data analysed across multiple groups were compared using an ordinary one-way anova (Graphpad 7.02, Prism). Data compared across two groups were compared using an unpaired *t*-test with Welch’s correction. Differences were considered significant when P < 0.05.

## Results

Six sheep were enrolled in this study. Only animals that survived for the duration of the study were included in the data analysis. Two animals had a pacemaker inserted and His bundle ablation during the same procedure. Of these, one animal died due to procedural complications and was therefore not included in the analysis. The remaining animals survived for the duration of the study and were included.

### Pacemaker devices were successfully implanted in all animals

**Fig 2:**
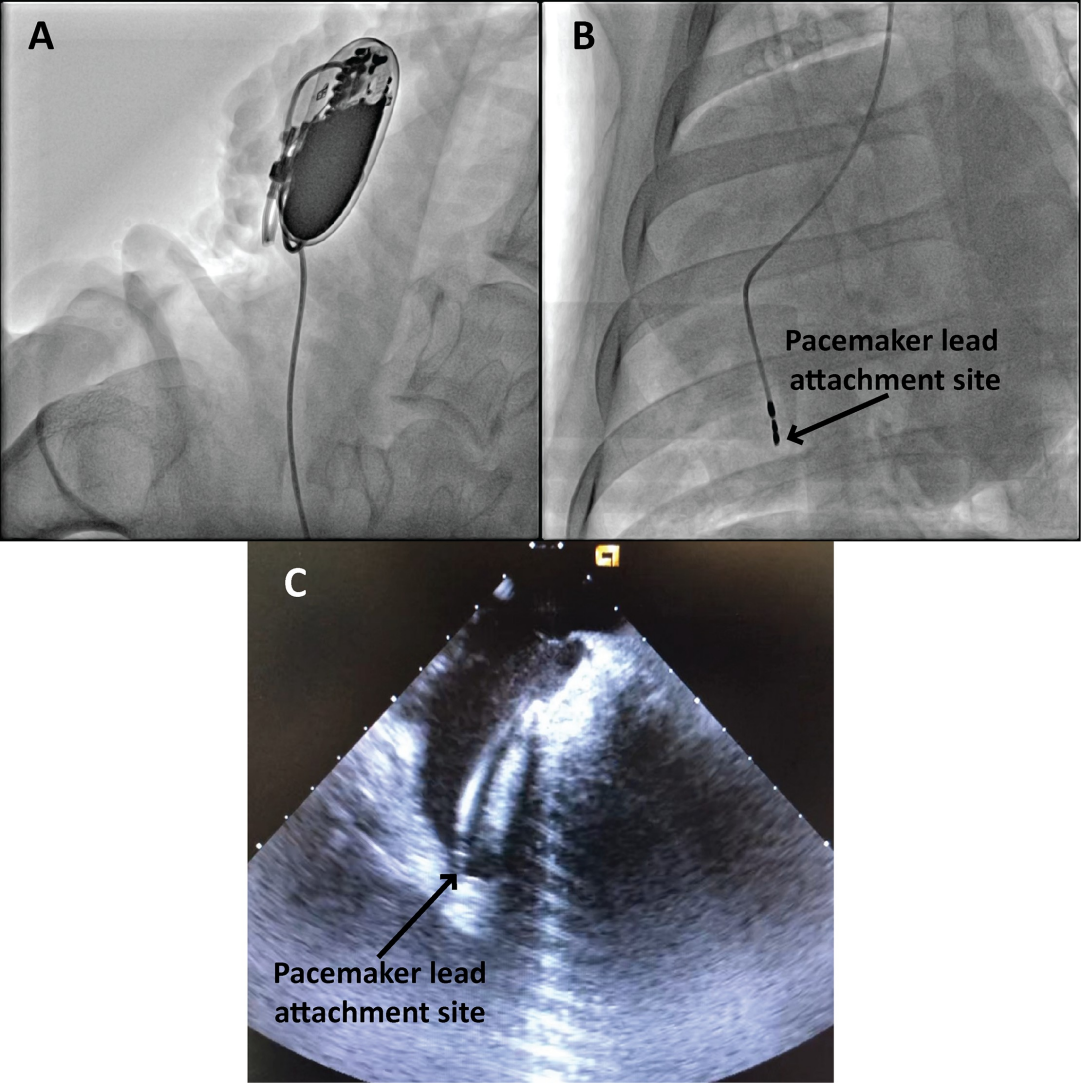
Representative fluoroscopy and ICE confirm anatomical location of the ventricular leads in the RV apex. (A) Fluoroscopic image demonstrating the anatomical location of the pacemaker device in the subcutaneous pocket. (B) Fluoroscopic image showing ventricular lead in the RV apex. (C) ICE image confirming position of the same ventricular lead in the RV apex.

Transvenous single chamber pacemaker implantation was successfully performed in all 6 sheep. Fig 2A shows a representative fluoroscopic image demonstrating the anatomical location of the pacemaker device in the subcutaneous pocket at the base of neck. Fig 2B is a representative fluoroscopic image showing the location of the ventricular lead at the RV apex for 4 of the 6 sheep. Positioning of the ventricular lead to the RV apex was confirmed via ICE as shown by the representative image in Fig 2C.

#### Variation in cardiac anatomy resulted in different lead attachment sites

**Fig 3:**
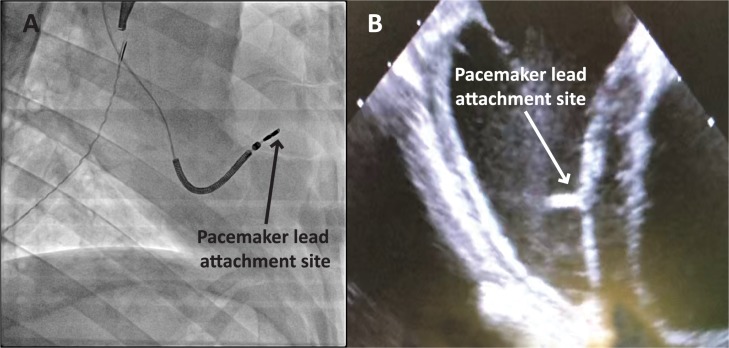
Representative fluoroscopy and ICE confirm attachment site of ventricular leads at the RV septal wall. (A) Fluoroscopic image showing the ventricular lead attached at the RV septal wall. (B) ICE recording confirming the ventricular lead attached at the RV septal wall.

The cardiac anatomy of animals enrolled in this study exhibited slight variations. Smaller ventricle size, large coronary sinus openings and less flexible leads meant two out of the six animals had the lead attached to the RV septal wall instead of the RV apex. This was confirmed by fluoroscopy (Fig 3A) and ICE (Fig 3B).

#### ECG and pacemaker diagnostics confirm pacemaker implantation

**Fig 4:**
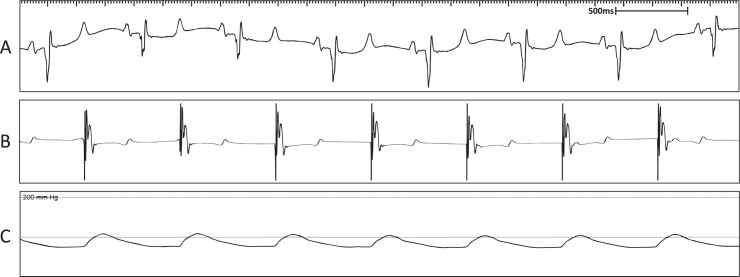
Representative surface ECG recordings during the pacemaker implantation to confirm lead placement. (A) Surface ECG depicting normal sinus rhythm under anaesthesia. (B) Surface ECG depicting paced rhythm during pacemaker diagnostics. (C) Mean arterial pressure trace indicating maintenance of blood pressure throughout the procedure.

Pacemaker functionality and lead placement was confirmed by successful capture of ventricular pacing as observed on ECG. Before pacemaker implantation, sinus rhythm was observed (Fig 4A). Lead placement was confirmed to be in the correct position by observing captured ventricular pacing on ECG after connection to the pacemaker device (Fig 4B). Arterial blood pressure remained stable throughout the procedure (Fig 4C).

### His bundle ablation successfully creates AVB model in adult sheep

Four weeks after pacemaker implantation and follow up monitoring, His bundle ablation and subsequent AVB model was successfully created in these 4 animals without any post-procedural complications. In a fifth animal, AVB was created although a fast, fascicular rhythm persisted for 3 weeks. A sixth animal died during the procedure due to procedural complications following creation of AVB. Experimental data and outcomes are summarised in Table 2.

**Table 2:** Summary of experimental data and outcomes of RF ablation experiments performed in sheep to create AVB.

Animal Number	Number of Ablations required	Complete AVB Achieved?	Follow up (weeks)	VF/VT during ablation	Notes
M8	2	Yes	0	VF Death	Mechanical AVB and VF Death following procedural complications
M9	23	Yes	4	Cardioverted VF Non-sustained VT	Fast fascicular rhythm competing with device for 3 weeks
M10	1	Yes	4	None	None
M11	12	Yes	2	Cardioverted VF	Cardioverted 4 times
M12	4	Yes	4	None	None
M13	13	Yes	4	Cardioverted VF	Cardioverted 7 times

**Fig 5:**
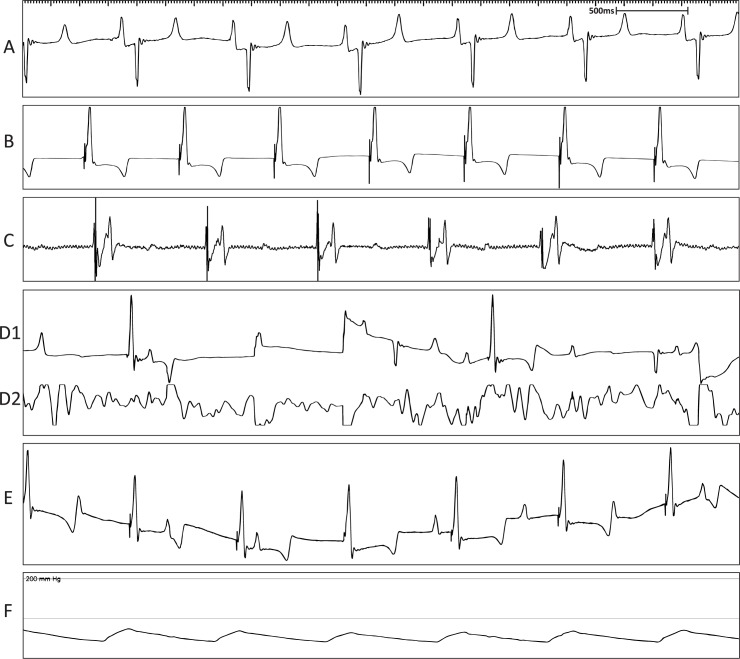
Representative surface ECG recordings during the ablation procedure confirming AVB model creation. (A) Surface ECG depicting normal sinus rhythm under anaesthesia. (B) Surface ECG depicting pacing rhythm during pacemaker diagnostics. (C) Bipolar electrogram from the ablation catheter proximal pair showing His electrogram before ablation. (D1) Surface ECG during ablation. (D2) Bipolar electrogram from the ablation catheter proximal pair during the ablation. (E) Surface ECG showing AVB evident by AV dissociation. (F) Mean arterial blood pressure trace indicating maintenance of blood pressure throughout the procedure.

**Fig 6:**
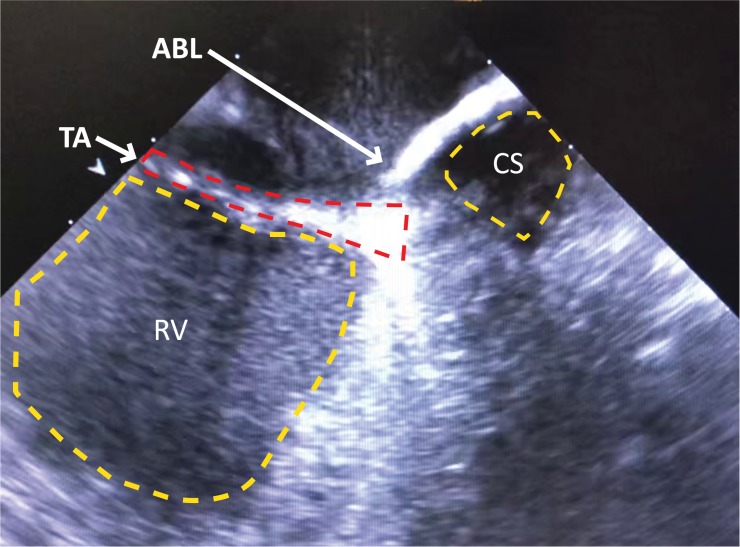
ICE recording confirms site tricuspid annulus (TA) and coronary sinus (CS) to assist with location of His bundle. Representative ICE recording confirming the location of the ablation catheter tip (ACT) at the site of the TA relative to the ostium of the CS and the right ventricle (RV).

For creation of AVB, a right atrial approach was used in all the animals. A regular surface ECG was obtained before the procedure indicating non-paced, normal sinus rhythm (Fig 5A). Pacing diagnostic tests undertaken before ablation confirmed pacemaker functionality (Fig 5B). ICE was used to anatomically define the tricuspid annulus and atrial septum (Fig 6). Endocardial bipolar electrograms from the ablation catheter localised the region of the His bundle by mapping for a typical His bundle electrogram (Fig 5C). RF ablation of the His bundle at this site was successfully performed (Fig 5D). All animals showed complete AVB (Fig 5E) as evidenced by AV dissociation and pacing reliance including preceding the death of sheep M8. All surviving animals showed persistent AVB, requiring constant pacing during an observational period of 4 weeks following ablation.

#### One animal exhibited a fast, fascicular rhythm following His bundle ablation

**Fig 7:**
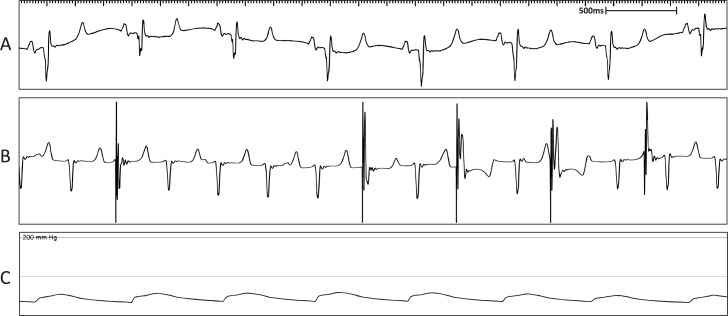
Representative surface ECG recordings following creation of AVB in sheep M9 showing a fast, fascicular rhythm. (A) Surface ECG depicting normal sinus rhythm. (B) Surface ECG showing AVB evident by AV dissociation with intermittent pacing due to the presence of a fast, fascicular rhythm competing with the pacemaker programmed at 80 bpm. (C) Mean arterial blood pressure trace indicating maintenance of blood pressure during the procedure.

All animals began with normal sinus rhythm (Fig 7A). In the case of sheep M9 however, although AVB was created in this animal, the rate generated by this escape rhythm competed with the device programmed at 80 bpm (Fig 7B). Although there was pacemaker dependence due to successful AVB, pacing reliance slowly increased from 85% to 100% over a 3-week period due to the fascicular rhythm dissipating.

#### Sheep are highly susceptible to ventricular arrhythmia during RF ablation of the His bundle

**Fig 8:**
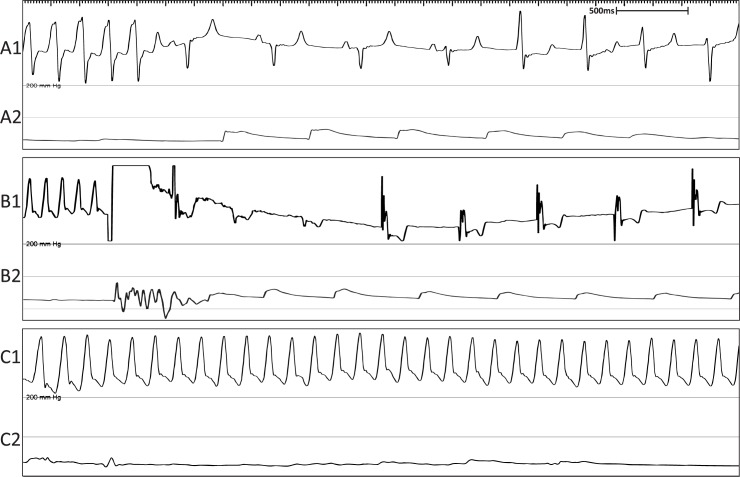
Representative surface ECG recordings showing VT and VF experienced during the ablation procedure. (A1) Surface ECG showing non-sustained VT. (A2) Mean arterial blood pressure trace indicating return of normal blood pressure following termination of VT. (B1) Surface ECG showing cardioverted VF. (B2) Mean arterial blood pressure trace indicating return of normal blood pressure following cardioversion of VF. (C1) Surface ECG showing VF causing death. (C2) Mean arterial blood pressure trace showing no blood pressure during VF.

Sheep undergoing His bundle ablation were highly susceptible to developing VT and VF, especially if the ablation site encroached into the RV. Four out of 6 animals experienced either non-sustained VT (Fig 8A) or cardioverted VF (Fig 8B). Only 1 animal died from sustained VF which could not be successfully cardioverted Fig 8C).

#### No ventricular escape rhythms following creation of AVB model in all animals

**Fig 9:**
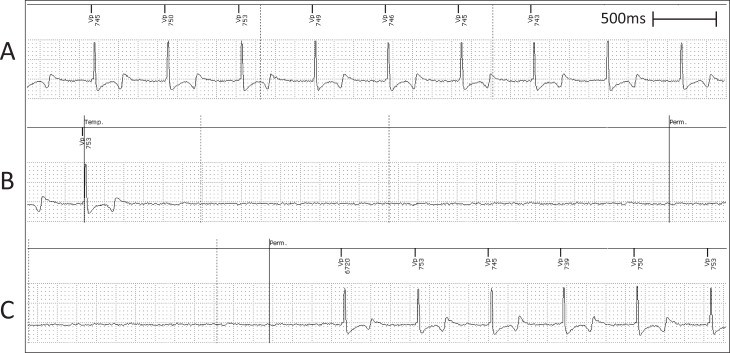
Lack of escape rhythm in the post-ablation follow up period. (A) Representative intracardiac ventricular lead recording depicting ventricular paced rhythm. (B) Representative lead recording showing lack of sinus rhythm when sensing feature activated by the pacemaker. Sensing period between the Temp. and Perm. lines. (C) Representative lead recording depicting return of ventricular pacing rhythm when sensing feature deactivated at Perm. line.

During the 4-week period following His bundle ablation, all animals were subjected to pacemaker sensing tests to determine the presence of ventricular escape rhythms. Fig 9A is a representative intracardiac ventricular pacemaker lead recording depicting the ventricular paced rhythm before the test. Once the sensing test was initiated, no ventricular escape rhythms were detected via the intracardiac lead (Fig 9B). The reappearance of the ventricular paced rhythm was due the to the termination of the sensing test and return of ventricular pacing (Fig 9C). The evidence of hemodynamic collapse during the sensing test confirmed pacing reliance due to successful creation of the AVB model. Further, all animals exhibited no evidence of ventricular escape rhythms at the 4-week time point, indicating sustained AV block.

### Analysis of diagnostic pacemaker data confirmed successful AVB model in adult sheep

**Fig 10:**
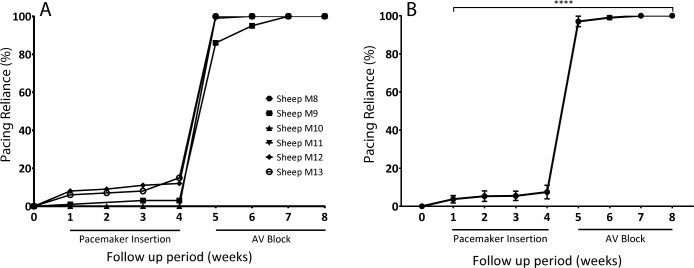
Diagnostic pacing data collected from pacemakers during baseline and post-ablation periods. (A) Pacing reliance was collected for 4 weeks following pacemaker implantation. His bundle ablation was performed at the week 4 timepoint, after which pacing reliance was collected for a further 4 weeks (B) Mean pacing reliance trends presented over the total examined period (% pacing reliance). Data is presented as mean ± SEM. N = 5. Statistically significant if p<0.05.

Five animals were monitored over 4 weeks for collection of baseline data and monitored for a further 4 weeks for collection of experimental data after His bundle ablation (Fig 10A). Baseline data across weeks 1–4 showed pacing reliance <8%. Once AVB was created, experimental data across weeks 5–8 showed a significant increase in pacing reliance to >95% (p<0.0001) (Fig 10B). When assessed in terms of time periods pre- and post-ablation, the pacing reliance significantly rose from 5.53 ± 1.28% to 98.89 ± 0.81% respectively (p<0.0001).

### Post-mortem analysis of lead attachment and ablation sites

**Fig 11:**
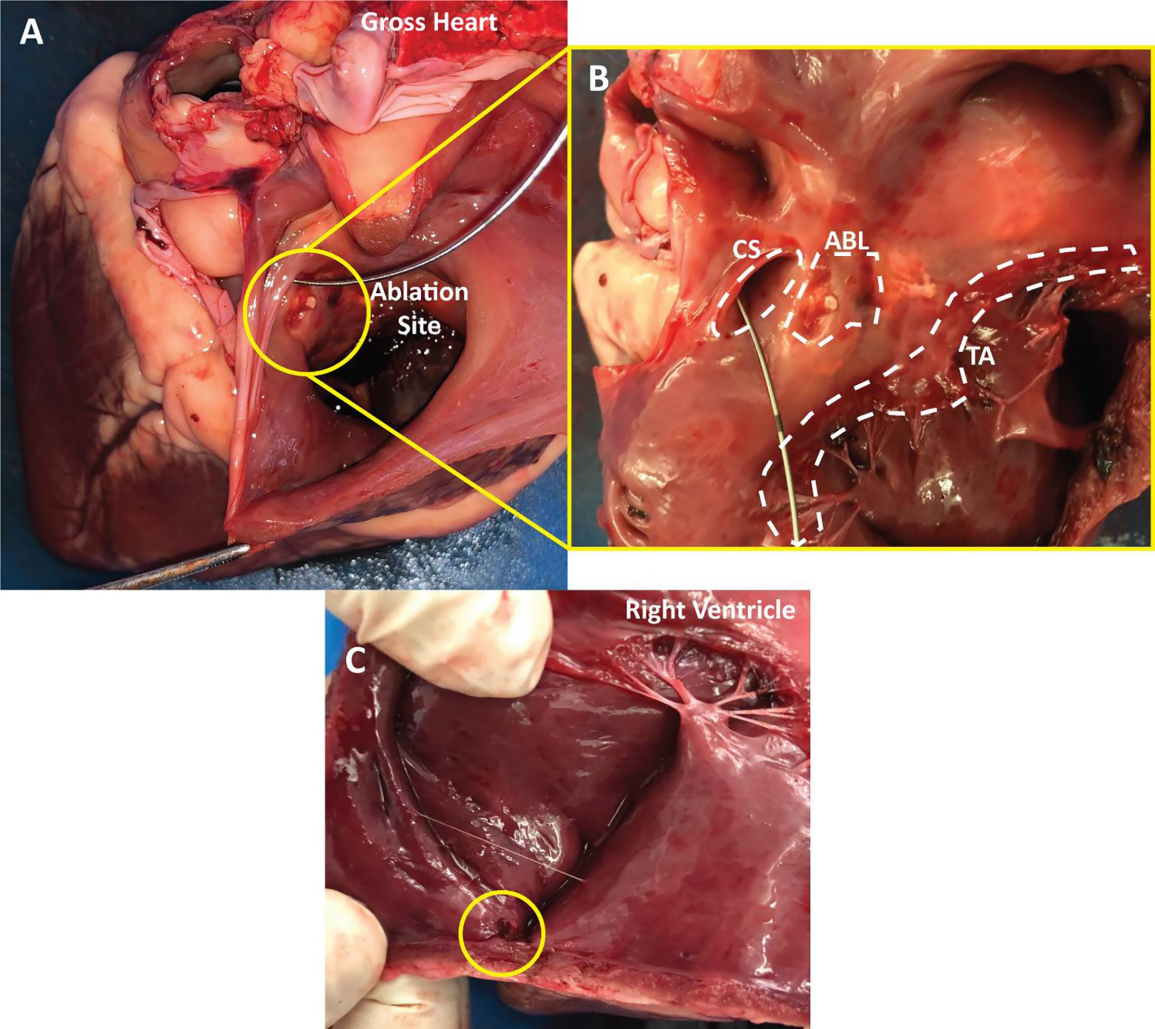
Gross anatomy of an explanted sheep heart showing landmarks, ablation lesions and lead attachment site. (A) This view depicts the gross heart anatomy with the view looking into the right atrium. The ablation site is shown within the circle. A wire was placed in the coronary sinus for orientation purposes. (B) Zoomed in perpendicular view of the coronary sinus (CS), tricuspid annulus (TA) and the ablation lesions (ABL). (C) View showing the lead attachment site within the circle at the apex of right ventricle.

Post-mortem analysis of gross heart specimens (Fig 11A) confirmed the endocardial location of the His bundle ablation site, favourably compared to fluoroscopy and ICE imaging. The ablation site was located at the mid atrial septum just superior to coronary sinus ostium (Fig 11B). The location of the lead attachment site was also confirmed to be in the RV apex as determined by fluoroscopy and ICE (Fig 11C).

## Discussion

In this study, we successfully developed, characterized and validated a large animal model of AVB using a conventional catheter-based, closed-chest His bundle ablation procedure that is stable, technically feasible and safe in adult sheep. To perform these experiments, we first inserted a single chamber, ventricular pacemaker device and then approached the His bundle from the right side of the heart and performed RF ablations at the site which demonstrated the largest endocardial His electrogram in conjunction with fluoroscopic and ICE guidance. These imaging modalities facilitated rapid lead and catheter placement and helped to avoid cannulation of the coronary sinus. This significantly streamlined the experimental procedure and minimised the procedural time, decreasing the risk of adverse outcomes. This resulted in a persistent, reliable AVB model requiring complete pacemaker reliance in the follow up period, which was significantly greater than the baseline period.

In earlier studies, Sill *et al*. successfully developed a paediatric sheep model of complete heart block using an atypical approach from the left side of the heart [12]. Bru *et al*. demonstrated the feasibility of His bundle ablation in adult sheep involving the delivery of very high RF energy to the tricuspid annulus [14]. Killsworth *et al*. utilized RF catheter ablations as a way of producing AVB for the assessment of bradycardia-induced spontaneous tachyarrhythmias and sudden death in conscious sheep following chronic myocardial infarction [13]. Although all studies successfully created AVB in sheep hearts, these studies did not stringently characterize and examine the pre- and post-ablation timepoints to confirm the successful creation of the AVB model, especially confirming the non-existence of escape rhythms or recurrent AV node conduction. We therefore needed to monitor the sheep over 4 weeks, to validate that our chosen method of creating AVB was successful. To use this animal model for the study of cell and gene therapy-based approaches in a pre-clinical context, we needed to be completely confident that potential results generated were originating from the treatment as opposed to escape rhythms or re-established AV node conduction from a poorly created model.

Sheep were chosen for this study because they are good models of cardiac anatomy and physiology [10]. Further, our research group has over 10 years of experience working with sheep as an animal model, boosting the reliability of our technical skill set and ability to successfully navigate creating this model. In our study, the first and only sheep died during the experiment due to procedural complications. A tremendous amount was learnt with regards to the differences between the anatomy of humans and sheep and how to better approach the development of this model with future animals. The two anatomical features which stood out included the smaller right ventricular size, and the increased size of the coronary sinus, resulting in difficulty manoeuvring the stiffer of the ventricular leads into the RV apex without repeatedly cannulating the coronary sinus [15]. This therefore meant that a subset of the animals had the lead attached to the septal wall as opposed to the ventricular apex (Fig 3). Although not in the ideal position, pacemaker diagnostics confirmed excellent pacing capture not affecting the functionality of the device.

A traditional right atrial approach assisted by fluoroscopy was used in all the animals to create AVB. This approach together with the use of equipment readily available for humans, mirrors the clinical approach used to create AVB in patients. ICE was additionally employed to streamline the process of positioning the ablation catheter at the tricuspid annulus, permitting easier identification of the His bundle for RF ablation. Varying numbers of ablations were required to achieve complete AVB in each animal, speaking to the variability in the exact location of the His bundle, the size of the ablation lesion required to interrupt conduction and the complexity of creating such a model.

Sheep were found to be highly susceptible to VT and VF during the ablation procedure especially if the ablation site encroached within the RV [14]. In 4 out of the 6 animals (66%), non-sustained VT, VT which lead to VF and VF requiring cardioversion were observed. These arrhythmias were transient, occurring during or immediately following the application of RF energy but only lasting a few minutes before self-termination or termination by DC shock. Only one animal died due to sustained VF, with no other animals displaying adverse events either during or following the procedure. The exact reason as to why sheep are more susceptible to VT and VF when ablations occur within the RV is still unknown. This observation means that future ablations will need to be performed at the atrial end of the His bundle to avoid ventricular tissue and minimise arrhythmic complications. The high rate of ventricular arrhythmias also means that equipment and personal need to be on standby for rapid defibrillation to promptly terminate potentially fatal arrhythmias.

One phenomenon that arose however was that of a fast, fascicular escape rhythm once the His bundle was ablated. This was transiently observed in several animals during ablations but only persisted for 3 weeks in one animal. We empirically determined that if a fast, fascicular rhythm was observed on ECG immediately following ablation of the His bundle, it was usually an indicator of unsuccessful AVB, with sinus rhythm re-emerging shortly thereafter. With the fast, fascicular escape rhythm however, pacemaker dependence was determined to be less than 100% at the first follow up as the escape rhythm competed with the pacemaker device programmed at 80 bpm (Fig 10). In the context of cell and gene-therapy based approaches, this outcome would not be acceptable as it would confound the result from the biological pacemaker. The outcome of new treatment options in pre-clinical assessment requires a sound baseline model. Although the rapid escape rhythm eventually dissipated and 100% pacing was observed at the 3-week timepoint, animals which do not have AVB and 100% pacing dependence at the time of treatment may need to be excluded.

Two further assessments were used to validate the creation of the model including: the percentage pacing reliance and the underlying presence of ventricular escape rhythms. An intrinsic feature of electronic pacemakers is the ability to record and store all parameters and readings associated with the device. One such measure is percentage pacing. This is a measure of how often the pacemaker is pacing the heart as a fraction of total time, presented as a percentage. This is therefore a measure used to quantify pacing reliance. As highlighted in Fig 10, all sheep showed a baseline reading of <8%. Ideally this would be 0% as there should be no pacing reliance before implantation. However, a programmed rate of 60bpm meant physiological changes in the heart rate–especially during the nocturnal periods–dipped below 60bpm, activating the device based on the programmed parameters. Although not ideal, it confirmed non-reliance on the electronic device and as such left a large margin with which to compare the pre- and post-ablation time points. Once the ablation was performed, pacing reliance increased significantly to 100% for all sheep except sheep M9. Fig 10A showed that sheep M9 started at 85% pacing reliance due to a fast, fascicular rhythm post-ablation. This rhythm was not persistent long term and as such, as the weeks progressed, the intrinsic rate slowed, and pacing reliance increased to 100% two weeks later. Concurrently, the sensing feature of the implanted devices allowed us to measure the presence of escape rhythms from a ventricular origin, through the pacing lead. All 5 animals showed no presence of escape rhythms at the 4-week timepoint including the animal which exhibited the fast, fascicular rhythm (Fig 9). This confirmed the creation of successful AVB with no ventricular escape rhythm, further validating our model.

Post-mortem analysis was performed on a subset of gross heart specimens to confirm that the location of the endocardial ablation and the lead attachment site conferred with the imaging modalities employed during the procedures. As highlighted in Fig 11, the ablation sites correlated with the fluoroscopic and ICE imaging, placing the location of the His bundle at the mid septum, anterior to the coronary sinus. Further, the coronary sinus was confirmed to be large, the RV being smaller in comparison to the whole heart and the orientation varying when compared with humans [10, 16]. Concurrently, the location of the lead attachment site was confirmed to be at the RV apex. We were therefore confident that the imaging modalities employed in tandem were providing an excellent representation of the internal anatomy, significantly assisting the procedural process.

There were limitations to the current study that can be addressed in follow up studies. For this study, a limited number of sheep were employed in the creation of this model. The sample size of six, although relatively small, showed creation of AVB with significant and complete dependence on pacing. Another limitation included sheep only being monitored for a relatively short period of 4-weeks following ablation. A disadvantage of AV dyssynchrony encountered with this model–because of RV only pacing–is the potential for cardiac dysfunction and cardiomyopathy. There is a body of work that shows single chamber ventricular pacing and AVB can lead to increased risks of atrial fibrillation and decreased quality of life [17–19]. It is unknown what the longer-term effects of our model are and whether this could affect the outcomes of experiments assessed over a longer period of time. However, treatments including the development of biological pacemakers using TBX18 have been shown to prevent and negate such these effects [20], building upon the longer term outcomes of these experiments.

## Conclusion

We have successfully developed, characterized and validated a large animal model of AVB that is stable and technically feasible in adult sheep. This model will allow for advancement of novel cell and gene therapy-based therapies, focusing on the development of biological pacemakers because we are confident that the model performs as designed, with the appropriate checkpoints in place to exclude animals which do not meet eligibility criteria for future work.
